# Happy and angry human pictures differentially affect dogs’ postural stability

**DOI:** 10.1038/s41598-026-37571-2

**Published:** 2026-02-03

**Authors:** Nadja Affenzeller, Christiane Lutonsky, Masoud Aghapour, Christian Peham, Barbara Bockstahler

**Affiliations:** 1 Physical Therapy, Clinical Centre for Small Animal Health and Research, Clinical Department for Small Animals and Horses, University of Veterinary Medicine (vetmeduni), Vienna, 1210 Austria; 2 Behavioural Medicine, Clinical Centre for Small Animal Health and Research, Clinical Department for Small Animals and Horses, University of Veterinary Medicine (vetmeduni), Vienna, 1210 Austria; 3 Movement Science Group, Clinical Centre for Equine Health and Research, Clinical Department for Small Animals and Horses, University of Veterinary Medicine (vetmeduni), Vienna, 1210 Austria

**Keywords:** Posturography, Center of pressure, Affective arousal, Postural stability, Dog, Emotion, Neuroscience, Physiology

## Abstract

In humans, visual stimuli are known to elicit biomechanical balance responses, thereby influencing postural stability. These responses offer valuable insights into the relationship between visual perception and physical balance. This is the first study to examine the effect of human pictures on postural stability in dogs. Seventeen healthy pet dogs were evaluated standing on a pressure plate under three conditions: viewing happy human pictures, angry human pictures, and no-picture. Five conventional Center of Pressure (COP) parameters were analyzed; mediolateral displacement, craniocaudal displacement, support surface, average speed (AS) and statokinesiogram length. Although no significant differences in balance were found across the three conditions overall, a cluster analysis of relative COP changes (ΔCOP_%, increase/decrease relative to the no-picture condition) revealed two distinct reaction patterns within both picture conditions. For happy pictures, 35% of dogs exhibited increases across all ΔCOP_% parameters (cluster H2), while 65% showed decreases (cluster H1). Angry pictures led to increases across all ΔCOP_% parameters in 47% of dogs (cluster A2), while 53% showed decreases (cluster A1). Comparisons within clusters H1/H2 and A1/A2 revealed significant differences in ΔCOP_% parameters (ANOVA, all ΔCOP_% parameters, *p* < 0.05, except AS within happy condition). No significant differences in ΔCOP_% parameters were found between clusters H1/A1 (decrease in all ΔCOP values) and between clusters H2/A2 (increase in all ΔCOP values). This exploratory study is the first to suggest that emotional human pictures evoke varied postural responses in dogs, influenced by the type of stimulus (happy or angry) and individual differences in emotional reactivity and sensory processing. Both emotions (happy, angry) resulted in increases in all ΔCOP_% parameters, interpreted as a destabilizing effect on balance, and decreases in ΔCOP_% parameters, interpreted as stabilizing effect on balance.

**Supplementary Information:**

The online version contains supplementary material available at 10.1038/s41598-026-37571-2.

## Introduction

Social cognition refers to the ability of an animal to perceive, recognize, evaluate and respond to social signals from conspecifics or other species^1^. In domestic dogs (Canis familiaris), this cognitive domain has developed due to the shared environment with humans, resulting in advanced skills for interpreting human social cues such as gestures, vocalizations, and facial expressions^2,3^. An emerging area of research in canine social cognition involves the use of screen-based paradigms where pet dogs are presented with two-dimensional facial images of humans. This method provides insights into dogs’ visual processing and affective understanding of human emotional expressions. Several studies have demonstrated that dogs can differentiate between emotional expressions in human faces^4^ and can integrate information across sensory modalities e.g., when simultaneously presented with human faces and human voice recordings^5^. Despite earlier concerns about whether dogs can interpret two-dimensional representations of human faces, accumulating evidence supports that dogs can recognize and respond to picture stimuli^6,7^, validating the use of facial pictures in experimental designs.

Emotional expressions in such stimuli can be conceptualized along the dimensions of valence and arousal, which describe the quality (positive–negative) and intensity (low–high) of an emotional state^8,9^. Beyond classification, valence and arousal also reflect motivational and adaptive functions of affective states, providing a useful framework for emotion assessment across species^10,11^. Parallel to this, there is growing interest in how emotional stimuli may influence not only perceptual and cognitive responses but also motor outputs such as postural stability. Postural stability is a fundamental motor function that enables both humans and animals to maintain balance and prevent falling through coordinated muscle activity working against gravity. Even standing still is an active, controlled process, governed by the integration of sensorimotor signals^12,13^. While visual (e.g., eyes closed vs. eyes open), proprioceptive, and vestibular inputs are traditionally recognized as primary contributors to balance control, recent studies have revealed the significant role of certain visual stimuli in modulating postural stability in humans^14,15^. Human research suggests that the effects of emotional pictures have been linked to postural changes, demonstrating balance shifts in response to both pleasant and unpleasant pictures, often interpreted as approach-withdrawal behaviour^16^. In addition, highly arousing stimuli can trigger freezing-like behaviours^15^.

Despite the known behavioural sensitivity of dogs to emotional human facial cues in pictures, there is limited research on how such stimuli may affect their postural control. While previous canine studies have focused primarily on behavioural and cognitive reactions to pictures, the biomechanical effects of emotionally salient visual stimuli remain underexplored. Standardized methods like posturography offer a robust tool for evaluating postural control by analyzing the displacement of the center of pressure (COP) within the base of support (BOS) of a subject. BOS is the area outlined by the contact points between the body and the supporting surface. The area in which the COP can move without the need for protective steps reflects the limits of stability^17,18^. Posturographic measurements during walk (dynamic) or quiet standing (static) are based on the measurement of ground reaction forces (GRF)^19,20^. The GRF represent the sum of all forces between a physical object and its contact surface. The COP is the point at which the instantaneous vector of the GRF is applied^13^. The displacement of the COP is an indirect measure of the functionality of postural control and thus, a measure of the ability to maintain balance, where in general smaller COP displacements are associated with superior postural stability^21^. Conventional COP metrics, including craniocaudal (CCD, anterior posterior in humans) and mediolateral displacement (MLD), total length of the COP (L) excursion, average speed (AS), and support surface (SS) provide indirect measures of balance stability^12,22,23^.

This study aims to bridge these domains by investigating the impact of emotional visual stimuli—specifically, pictures of happy and angry human faces—on postural stability of dogs during quiet standing. By employing posturographic analysis across three visual conditions (happy face, angry face, and no-picture), we seek to explore the interplay between emotional visual perception and biomechanical balance responses in dogs. Findings from this research may not only advance our understanding of affective influences on motor control in non-human animals but also contribute to broader insights into the co-evolution of emotional communication and sensorimotor integration in the dog-human relationship.

## Results

The means of all 5 COP parameters of two valid trials per condition were analyzed for all 17 dogs included in this study; the mean duration of viewing the happy picture was 4.9 ± 0.2s, for the angry picture 4.9 ± 0.1s and for the no-picture condition 5.0 ± 0.0s. In the PANAS questionnaire, which assesses a dog’s sensitivity to both rewarding and aversive stimuli (for more details see^24^, all dogs scored near the reported median of 0.68 and within the interquartile range of 0.19 for positive activation. Scores ranged from 0.66 (dog 3) to 0.86 (dog 10). Negative activation scores varied between 0.24 (dogs 5 and 7) and 0.76 (dog 12), with the published median at 0.44 and an interquartile range of 0.26. Individual results for each dog are provided in Table S1.

Of all valid trials, 63% (*n* = 43) were collected during the first session, 24% (*n* = 16) during the second session, and 13% (*n* = 9) during the third session. In total, 46% of valid trials were analysed while dogs viewed male faces, and 54% while viewing female faces. In trials involving happy facial expressions, 44% of valid responses were analysed from male faces and 56% from female faces. Similarly, for angry facial expressions, 44% of valid trials corresponded to male faces and 56% to female faces. Detailed information for each individual dog is provided in Table S1.

Based on the ANOVA analysis, no significant differences were found between all 5 COP data (MLD_%, CCD_%, L_%, AS, SS_%) when comparing all three conditions (Table 1). In addition, no significant differences were found between all non-normalised COP data (MLD, CCD, L, SS), BOS L and BOS W when comparing all three conditions (Table S2).

**Table 1:** Descriptive statistics (mean values ± SD) of all COP_% parameters in different visual conditions.

Condition	MLD_%	CCD_%	L_%	AS	SS_%	BOS L	BOS W
No-pic	1.25 ± 0.33	1.16 ± 0.26	0.09 ± 0.03	20.43 ± 6.83	0.08 ± 0.03	55.45 ± 7.48	19.92 ± 2.67
Happy	1.27 ± 0.36	1.08 ± 0.22	0.08 ± 0.02	18.56 ± 4.09	0.08 ± 0.03	54.39 ± 6.30	21.09 ± 3.67
Angry	1.34 ± 0.43	1.18 ± 0.36	0.08 ± 0.02	17.94 ± 4.40	0.09 ± 0.04	53.35 ± 6.42	21.80 ± 3.80

MLD_%: mediolateral displacement (%); CCD %: craniocaudal displacement (%); L_%: length of the COP (%); AS: average speed of the COP in mm/s; SS_%: support surface (%); BOS L: distance between the center of the front and hindlimbs in centimeters (cm) ; BOS W: distance between the center of the left and right limbs in centimeters (cm); ±: standard deviation; no-pic: no-picture presentation; happy: happy human picture presentation; angry: angry human picture presentation.

When analyzing the individual reactions of all dogs (ΔCOP_%, increase/decrease relative to the no-picture condition) in all COP data, large differences were observed. The smallest difference was ΔL_% and ranged from a -57.0% decrease (dog 11, happy picture) to 64.1% increase (dog 14, happy picture), comprising a total range of 121.2%. This was closely followed by a total range of 122.8% of ΔAS and 123.1% of ΔCCD_%. The highest difference was ΔSS_% and ranged from − 67.7% (dog 11, happy picture) to 186.4.% (dog 2, angry picture), comprising a total range of 254.1%. This was closely followed by ΔMLD_% were a total range of 181.5% was observed. Details on individual dogs and their absolute and relative differences of all COP parameters including their cluster numbers are presented in supplements Table S3.

For both visual conditions (H, happy; A, angry) inspection of the Hierarchical Cluster analysis dendrogram supported a 2-cluster solution (1, 2) in both conditions. Subsequently, a *k*-means cluster analysis (*k = 2*) identified two distinct clusters in both visual conditions (happy, angry) (see Table 2). Box plot graphs depicting all ΔCOP_% data between the clusters are presented in Fig. 3. ANOVA analysis confirmed significant differences between both identified clusters (1 vs. 2) in all ΔCOP_% parameters for both visual conditions H and A (except for ΔAS, condition happy, *p* = 0.50).

**Table 2:** Results of K-means cluster analysis based on visual condition.

Cluster	Condition angry		ANOVA		Condition happy		ANOVA	
	A1	A2	F	*p*	H1	H2	F	*p*
ΔMLD_%	-17.09	52.77	15.73	*0.001	-15.20	51.65	18.63	*<0.001
ΔCCD_%	-13.13	25.68	9.27	*0.008	-15.61	18.96	16.05	*0.001
ΔL_%	-24.62	3.00	9.55	*0.007	-16.46	12.02	5.01	*0.04
ΔAS	-18.93	5.43	7.33	*0.016	-6.19	3.63	0.49	0.50
ΔSS_%	-27.26	83.03	30.08	*<0.001	-23.48	52.29	35.03	*<0.001
Dogs	9	8			11	6		

Cluster: cluster number (1, 2, A – angry, H- happy, ) based on K-Means cluster analysis; ΔMLD_%: relative mediolateral displacement (%); ΔCCD %: relative craniocaudal displacement (%); ΔL_%: relative length of the COP (%); ΔAS: relative average speed of the COP (%); ΔSS_%: relative support surface (%); dogs: number of dogs within the identified cluster; condition happy: happy human picture presentation; condition angry: angry human picture presentation; F: F-test value; *p < 0.05 statistically significant.

When viewing angry pictures, dogs grouped in cluster A1 showed an overall decrease in all 5 ΔCOP parameters (53% of dogs, *n* = 9) whereas dogs grouped in cluster A2 (47% of dogs, *n* = 8) predominantly increased in all ΔCOP parameters. When viewing the happy pictures, dogs grouped in cluster H1 (65% of dogs, *n* = 11) showed an overall decrease in all 5 ΔCOP parameters, whereas dogs grouped in cluster H2 (35% of dogs, *n* = 6) predominantly increased in all 5 ΔCOP parameters (for details see Table 2).

When comparing all ΔCOP_% parameters of dogs based on their cluster number, ANOVA analysis showed no significant difference in all 5 ΔCOP parameters between both clusters H1 and A1 (predominantly decreased values) and between both clusters H2 and A2 (predominantly increased values).

## Discussion

Visual stimuli are known to induce biomechanical balance responses, influencing postural stability in humans^14–16^. This exploratory study for the first time investigated the effects of emotional visual stimuli, specifically viewing happy and angry human pictures, on postural stability in pet dogs during static stance. The emotional arousal when viewing human pictures had both, a stabilizing and destabilizing effect on balance in dogs.

When analysing the three visual conditions no significant differences were observed in any COP parameters. However, based on canine behavioural research studies pet dogs share certain behavioural strategies, especially when confronted with situations that are perceived as threatening. Known behavioural reactions include fight, flight, freeze and fidgeting responses^25–27^. This means, that individual dogs may react with opposing strategies when confronted with the same stimulus. For example, while one dog might move away a different dog might move towards the same stimulus. Indeed, in a human study, personality traits and individual psychological characteristics predicted threat-induced changes in postural control^28^.

To explore the variability in responses, COP data were also analysed at the level of individual dogs, revealing significant differences in their reactions. While some dogs exhibited a marked increase in COP values, others showed a decrease. The extent of these individual variations was most pronounced in ΔSS_% (range: 254.1; minimum: -67.7%, maximum: 186.4%) and least pronounced in ΔL_% (range: 121.2; minimum: -57.0%, maximum: 64.1%). SS serves as a comprehensive measure of postural performance, accounting for 90% of the two-dimensional overall direction of COP excursions (mediolateral and craniocaudal), typically forming an ellipse that represents the sway area^23^. As such, it directly reflects an individual’s ability to maintain their center of mass within the BOS. Unsurprisingly, SS is influenced by task complexity, surface conditions, and the individual’s balance capabilities in humans^29,30^. Conversely, the smallest variation was observed in ΔL_%. Research in humans has shown that total length of the COP excursion is influenced by the general ability to maintain stability rather than reflecting specific postural control mechanisms^31^. A similar result was observed when comparing old and young dogs when challenged by standing still when blindfolded: despite significant age-related differences in SS_%, both groups maintained a relatively consistent L_%^32,33^. Therefore, the parameter L may not be suitable for detecting effects of visual cues on postural control mechanisms.

Hence, we postulate that this difference in individual responses may explain the non- significant results when analyzing the three visual conditions on a group level. This variability observed in ΔCOP_% responses highlight the complexity of visual-emotional interactions, consistent with studies in humans demonstrating that emotional responses and postural adjustments vary significantly and could be based on individual sensitivity and prior life experiences^34,35^. Indeed, grouping the dogs using cluster analysis revealed that 6 of 17 dogs in the happy picture condition and 8 out 17 dogs in the angry picture condition reacted with an overall increase in all 5 ΔCOP_% parameters. This finding implies a destabilizing effect on a subpopulation of dogs on balance, irrespective of the emotional valence of the picture. On the other hand, an overall decrease in all 5 ΔCOP_% parameters was observed in 11 out of 17 dogs when viewing the happy pictures and 9 out of 17 dogs when viewing the angry picture, implying a stabilizing effect on balance.

While emotional valence (positive or negative) is often emphasized in studies of emotional stimuli, evidence increasingly suggests that arousal, rather than valence, is the key determinant of postural responses^36^. For example, although human studies have demonstrated that unpleasant visual stimuli frequently elicit freezing behaviours, other studies report an increase of COP parameters to similar stimuli^16,37–39^. These findings align with our observation that both happy and angry pictures elicited ΔCOP_% changes.

Nevertheless, a more stable balance (an overall reduction in all ΔCOP_% parameters), might also be interpreted as freezing behaviour to a threat. However, none of the included dogs displayed any behavioural signs indicative of distress or fear while viewing any of the human pictures or while participating in the study (determined by the first author NA who is a Diplomate of the European College for Animal Welfare and Behaviour Medicine). It is important to note that maintaining a motionless posture, including absence of head and tail movements, during picture presentation was a necessary condition for a valid data collection. Consequently, behavioural stress indicators such as head turning, lip licking or paw lifting^25,26^ were not observed during data collection. Therefore, qualitative behavioural assessment alongside static posturography appears limited in its utility for detecting stress in dogs. Supporting this, a recent study employing qualitative behavioural assessment to evaluate short-term emotional states, considering both valence and arousal, found that only panting, whining, and body shaking correlated significantly with arousal, and exclusively in negatively valenced contexts. In contrast, increased activity and sitting behaviour were linked to positively valenced, high-arousal states^9^. Future studies could benefit from using the Dog Facial Action Coding System (DogFACS), a standardized and validated method for objectively categorizing facial muscle movements capturing unique canine facial expressions^40^, to more accurately assess welfare and emotional states of dogs. In this study, all dogs were considered emotionally healthy, which was supported using a PANAS questionnaire, specifically developed and validated for dogs. This questionnaire assesses emotional states by quantifying positive and negative affective traits, serving as indicators of each dog’s temperament^24^. This is why a perceived threat, specifically when viewing the happy pictures, appears rather unlikely. Despite all dogs standing completely still during the data collection, we cannot completely rule out a freezing response for the angry picture condition. Notably, freezing responses are not exclusive to negatively valenced stimuli in studies investigating balance in humans; similar reactions have been observed in response to positive stimuli^41^.

We propose that some of the observed postural responses, particularly in cluster H1 (overall decrease of COP parameters) in the happy picture condition, may reflect anticipatory strategies- a phenomenon known from human studies. Anticipatory postural adjustments (APAs) are defined as the activation of postural muscles in a feedforward manner to prepare for destabilizing forces associated with voluntary movement^42^. APAs are learned behaviours based on prior experiences of postural disturbances and serve to optimize stability during anticipated challenges^43^. Dogs that displayed predominantly decreased ΔCOP_% parameters may have engaged in preparatory postural adjustments when viewing happy pictures. This could be indicative of a learned response to manage the anticipated impact of emotionally salient happy visual cues, possibly influenced by the presence of their handler behind the screen or previous exposure to similar situations. Indeed, the stabilizing effects of happy pictures in cluster H1 of dogs may reflect attentional focus in anticipation of a positive interaction with the dog handler, rather than a behavioural freezing response in a perceived threat. To assess the dog handler as a potential confound, future studies should include conditions without familiar people being present in the room or behind the screen.

Several limiting factors must be considered when interpreting these findings.

No video-based eye tracking systems were employed in this study so that eye position and gaze direction could not be directly evaluated^44^. While experimenters visually ensured that the dogs’ heads and snouts were aligned toward the screen, with no head movement allowed during data collection, it is still possible that some dogs averted their gaze from the stimuli. Although all participating dogs were required to respond to a “wait in a standing position” cue, the study opted for a head-free setup rather than implementing a training protocol that required a fixed head position (e.g., resting the head on a surface to enable eye-tracking devices to monitor pupil-corneal reflection). This approach allowed the dogs to exhibit more natural behaviours such as avoidance behaviours like head turns or displacement and conflict behaviours (e.g., lip licking or yawning)^45,46^. Indeed, one dog was excluded when moving away with a lowered tail after viewing the first picture, a behavioural sign indicative of a fear response.

Importantly, dogs have a wider field of view (approx. 240° compared to humans’ 140°) due to their laterally positioned eyes^47^, making it challenging for them to completely avoid looking at the screen without moving their head in this setup. Additionally, skull morphology impacts on visual capabilities. While lateral eye positioning increases the field of view it reduces the binocular vision overlap. For this study, only dogs with mesocephalic head morphology, as determined by a veterinarian, were included. However, it is still unclear if and how differences in dogs’ visual system may impact visual perception of emotional cues^45^.

A key limitation of the current study is the absence of a neutral facial expression condition. While the design included happy and angry facial expressions the no-picture condition does not adequately serve as a control for the perceptual and social salience inherent in any human face, regardless of its emotional content. This omission restricts our ability to determine whether the observed postural responses are attributable to the emotional valence (positive vs. negative), arousal level, or the presence of a socially relevant stimulus. While we may conclude that these changes are not driven by valence alone (since similar patterns were seen in response to both happy and angry expressions), the absence of a neutral comparison picture makes it speculative to attribute them solely to arousal. Without a neutral facial reference, we cannot confidently rule out other contributing factors such as attention to facial stimuli or generalized social engagement. Thus, while our findings suggest a link between facial emotion perception and postural adjustments, conclusions regarding the specific role of arousal versus valence must be viewed with caution. Future research should incorporate a neutral human expression as a reference condition to better disentangle the contributions of valence, arousal, and face-specific processing. This would strengthen the interpretability of emotion-related changes on balance and provide a more rigorous test of emotion–action coupling in dogs.

Another aspect that we were unable to control was the focus of attention. The repeated passive presentation of only two human faces expressing two different emotions may have resulted in habituation and boredom, potentially influencing attentional processes^48^. To mitigate this, we limited the presentation of each stimulus to three times per category (female happy, female angry, male happy, male angry) and included a moving object on the screen along with a neutral sound between human picture presentation to help maintain or regain attention. Head movements directed toward the picture at the onset of the presentation or away from the picture at the end of the 5- second display were associated with reduced screen viewing duration (mean viewing times were 4.9 ± 0.2s for happy pictures, 4.9 ± 0.1s for angry pictures, and 5.0 ± 0.0s for the no-picture condition). Importantly, 63% of valid data were obtained during the first presentations round (trial 1) and 24% during the second round (trial 2), which likely helped reduce the influence of repeated exposure effects such as habituation and reduced arousal. However, since all trials were performed on the same day, this may have affected the dogs’ attentional capacities. To address this limitation, future studies could benefit from incorporating a greater variety of stimuli and spacing testing sessions across multiple days to better maintain attention and reduce fatigue.

The considerable individual variability seen in ΔCOP_% responses underscores the necessity for larger sample sizes. Because the same COP variables were used for both the clustering procedure and the subsequent statistical tests, these analyses should be viewed as exploratory and descriptive rather than confirmatory of potential postural response subtypes. Future studies should evaluate the quality and stability of the clusters and verify the cluster structure using an independent canine dataset.

Another limitation to consider involves influences like life stage (such as age and previous experiences) and individual temperament. Previous research has demonstrated that a dog’s ability to interpret human facial expressions is shaped by the quality and amount of exposure to human faces^48^, and that dogs are capable of distinguishing between familiar and unfamiliar faces^49^. In this study, only images of two unfamiliar individuals were presented to the dogs to minimize familiarity bias. However, given that pictures of only two human subjects were used, it is important to note that the findings may not fully generalize to dogs’ responses to a broader range of human faces. Additionally, it is possible that some dogs had greater exposure to human faces over their lifetimes than others, as their ages ranged from 1.4 to 6.3 years. These life experiences may also involve varying levels of positive or negative social interactions with humans, which could not be accounted for.

That said, all participating dogs were pet dogs living with their owners in urban environments, and none displayed behaviours indicative of conflict or social fear toward the experimenters. This suggests that, on average, the dogs likely had exposure to a variety of people. Nonetheless, even though all dogs were considered emotionally healthy and showed no behavioural signs of stress, individual differences in past experiences—both positive and negative—may have influenced their postural responses. Psychological traits or states may also contribute to the observed interindividual variability in the results. Future research should aim to better characterize factors such as an individual dog’s life history, personality, and experiences (in human, fear, trauma, etc.) to deepen our understanding of the relationship between movement, balance control, and emotional information processing.

Seventeen dogs completed all sessions and met the strict criteria for valid trial inclusion, which required standing still during stimulus presentation. While this sample size is consistent with existing studies employing similar methodologies^5,7,21,48–50^, we acknowledge that it remains relatively small and may limit both the statistical power and generalizability of the findings. Additionally, the sample size did not permit separate analyses based on either the sex of the participating dogs or the sex of the human faces shown (male vs. female). Consequently, potential sex-based differences in emotional or postural responses—whether related to the subjects themselves or the facial stimuli—could not be investigated within the scope of this study. Nevertheless, the gender distribution of participants (47% females, 53% males) and the gender distribution of human pictures (male vs. female and happy vs. angry ranged between 44% and 56%, see results for details) were evenly distributed in all analysis.

Our findings suggest that future studies should include physiological measures of arousal such as heart rate and heart rate variability measurements to gain a more comprehensive understanding of arousal and emotional valence. Previous research has shown that both happy and angry human faces can increase a dog’s heart rate, while sad human faces tend to decrease it^50^. Furthermore, the anticipation of the activity of chasing a lure for 50 m while watching other Greyhounds run has been shown to significantly increase heart rate^51^. Additionally, changes in heart rate variability have recently been associated with arousal levels in negative valenced stress scenarios^9^. Therefore, heart rate and heart rate variability could serve as useful physiological indicators for assessing arousal levels and the intensity of emotional responses^9^. Additionally, integrating electromyography data to capture muscle activity could provide more direct evidence for anticipatory motor adjustments behaviours, a topic that has yet to be explored in dogs.

Based on the findings discussed above, it can be tentatively concluded that viewing emotional human faces elicits postural responses, which may vary depending on individual temperament, influencing arousal levels and triggering different compensatory balancing mechanisms in the participants.

In summary, this exploratory work offers the first preliminary evidence that emotional human pictures evoke varied postural responses in dogs, influenced by the type of stimulus (happy or angry) and individual differences in emotional reactivity and sensory processing. This research offers new perspectives on the relationship between visual stimuli, emotional processing, and postural control in dogs, enhancing our understanding of sensory-emotional integration in non-human species.

## Methods

### Approval and consent

This study complied with ARRIVE guidelines and was discussed and approved by the institutional Ethics and Animal Welfare committee of Vetmeduni, University of veterinary medicine, in accordance with the University’s guidelines for Good Scientific Practice guidelines and national legislation (ETK-148/10/2021). Informed consent was obtained from all dog caretakers.

The human pictures were made available by Albuquerque Natalia^5^. Written informed consent was obtained from the human models whose pictures were used and who also agreed that their pictures can be used for research and related publications. All methods were performed in accordance with the Declaration of Helsinki and relevant guidelines and regulations. Ethical approval was granted by the ethics committee in the School of Life Sciences, University of Lincoln, UK^5^.

### Animals

A total of 25 client-owned healthy dogs were evaluated in this study allowing for an estimated drop out range of 20%. These numbers were based on previously published studies using facial images and their effects on dogs^5,7,48,49^ and veterinary studies on canine biomechanic using static posturography^21,52,53^. The inclusion criteria required an age < 50% of fractional lifespan (for details see data analysis), the absence of clinical musculoskeletal, neurological, or visual disease, and all dogs underwent a comprehensive clinical examination by qualified veterinarians including visual gait assessment, orthopaedic evaluation, and calculation of symmetry index (SI, for details see data analysis) when walking. Seven dogs were excluded due to their inability to remain still while exposed to the pictures. One dog moved away from the screen with behavioural signs indicative of fear (moving head away combined with lowering the tail and consequently walking away from the picture stimulus) and was excluded from further trials. The remaining dog breeds included Labrador retriever (*n* = 4), mixed breeds (*n* = 3), border collie (*n* = 3), Golden retriever (*n* = 2), Malinois, Irish terrier, pointer, greyster and a Flat coated retriever. The dogs’ body mass was from 13.5 to 36.0 kg (24.2 ± 5.8), and their ages ranged from 1.4 to 6.3 years (3.6 ± 1.4). Eight dogs were male (5 intact and 3 neutered), and 9 dogs were female (4 intact and 5 spayed). Dog height was from 0.46 m to 0.67 m (mean 0.56 m ± 0.06). Dog length was from 0.46 m to 0.67 m (mean 0.57 ± 0.06 m). All dogs were required to have more than 10 kg body mass and to have a mesocephalic skull morphology. All caretakers have previously completed basic obedience training with their dogs, including a “wait in a standing position”.

### Equipment and measurement procedure

In this prospective study, the COP parameters of the dogs were measured in a quiet standing position on a flat ground by using a Zebris platform (FDM Type 2, Zebris Medical GmbH, Allgäu, Germany) equipped with 15, 360 sensors covering an area of 203 × 54.2 cm and a measuring frequency of 100 Hz. The sensor size of the platform was 0.72 × 0.72 cm. To standardize the coefficient of friction, the pressure plate was covered with a black 1-mm-thick non-slip rubber mat made of polyvinyl chloride. All measurement procedures were filmed using a Panasonic NV-MX500 camera (Panasonic, Kadoma, Osaka, Japan) with a standardized setup for camera positioning and angle. The camera was positioned to capture the dog standing in front of a TV screen (40 inch, 1080p, full HD, 1920 × 1080, 24p, 100 Hz, brightness 500 cd/qm, Samsung, UE40B6000, Samsung Electronics Co., Ltd., Suwon, South Korea).

**Fig. 1:**
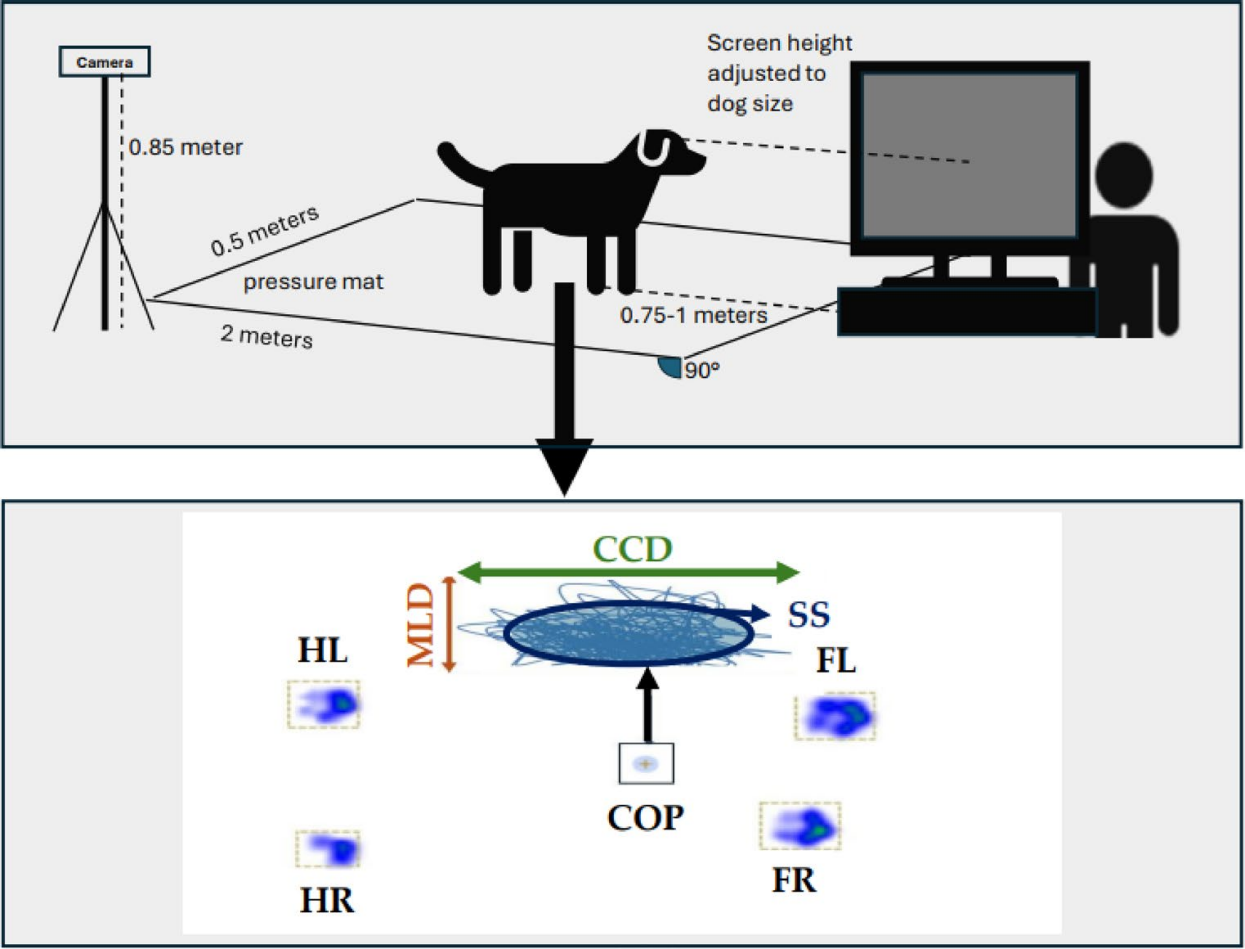
Visual images used in this study provided by^5^. Pictures presenting a male and a female drama student with happy (right hand side) and angry (left hand side) facial expressions.

Each dog was measured during 3 conditions, including visual conditions and a no-picture condition (blank screen). The visual stimuli were pictures of two adult drama students (1 female, 1 male) expressing either positive (happy) or negative (angry) emotions (Fig. 1). The no-picture condition did not include any visual nor auditive stimuli. These pictures have previously been used to demonstrate the ability of pet dogs to recognize human emotions and were generously provided by Albuquerque and her colleagues^5^. Pictures were generated with controlled lightning asking the models to evoke the emotions through their faces. To correct for facial color differences grey scale images were used to minimize contrast effects. The frontal face picture (3008 × 2000 pixels) had grey homogenous background and were gamma corrected and normalized for luminosity to account for brightness and contrast (for more details see^5^. The visual stimuli were presented in a random order featuring both happy and angry pictures from the female and the male student. Visual stimuli order in trial 1 was female happy, male angry, male happy, female angry, in trial 2 female angry, male happy, male angry, female happy and in trial 3 female happy, male happy, female angry and male happy. In between picture presentation a blue screen with two rotating yellow stars along with a neutral sound appeared over 2 s to help the dog maintaining its focus on the screen. Each visual stimulus was played once over 5 s per trial. Therefore, each dog was presented with each possible combination (male, female, happy, angry) once per trial. Each dog took part in three trials, resulting in a total viewing of the happy male picture 3 times, of the happy female picture 3 times, of the angry male picture 3 times, and of the happy female picture 3 times. The dogs were able to have a break between trials and received treats after each trial. To ensure that no psychological harm was introduced by viewing the stimuli, the behaviour of all dogs was permanently monitored by the experimenters (all veterinarians, one of them an animal behaviour specialist) to ensure that all dogs remained comfortable.

### Static posturography

To familiarize the dogs with the room, the set-up, and the equipment, they were allowed to wander around freely for up to 15 min. Once the dogs were familiarized with the room, GRF were calculated when walking in a straight line using the pressure plate mentioned above. At least five valid passes were analyzed to calculate the SI for peak vertical force (PFz) and vertical impulse (IFz). A valid pass was defined as a walk in which the dog crossed the plate without changing pace, turning its head, pulling on the leash, or touching the dog handler. The difference in speed at which the dogs crossed the plate had to be within a range of ± 0.3 m/s and an acceleration of ± 0.5 m/s^2^. The SI for PFz, and IFz of all included dogs in this study was below 3% which is the margin typically used to diagnose a lameness free gait pattern^53^.

**Fig. 2:**
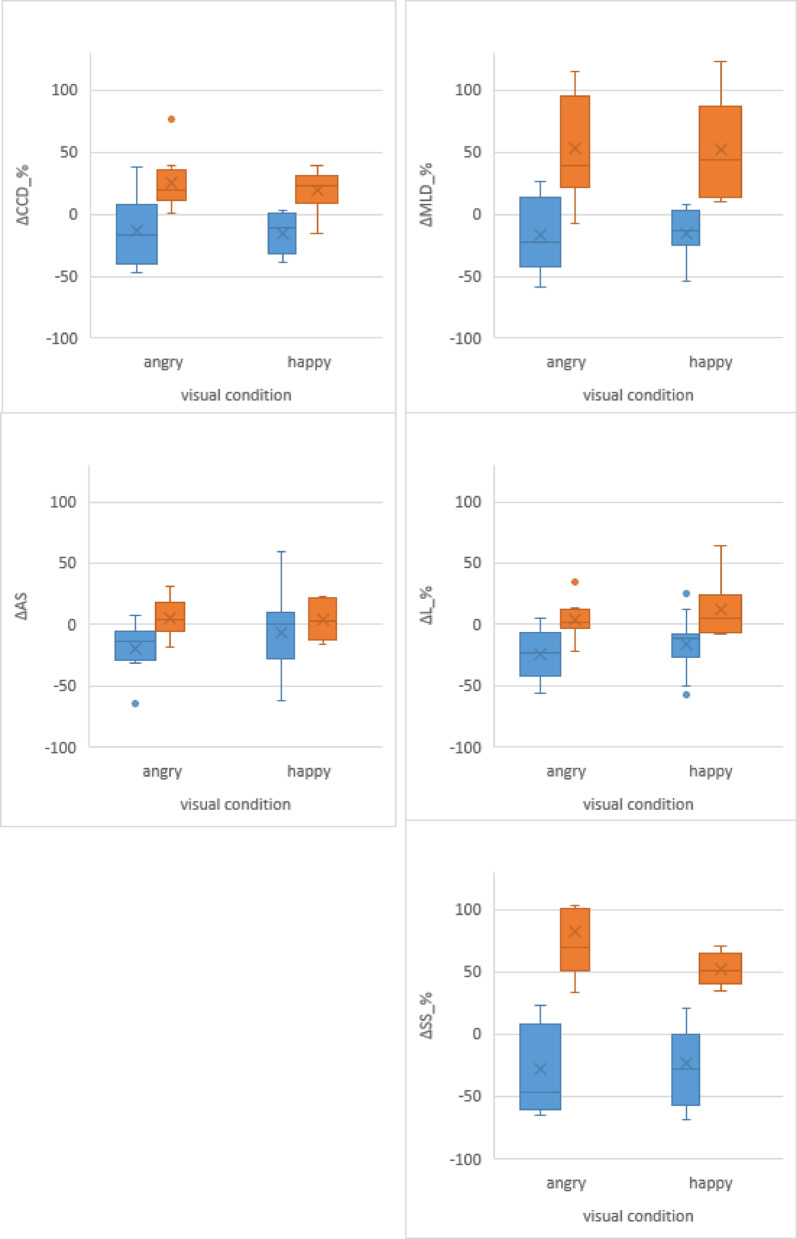
Schematic set up; the dog is positioned on top of the pressure mat facing the screen. The kneeling dog handler is hiding behind the screen. The camera is placed on top of a tripod and records each trial. CCD: craniocaudal displacement of the COP; COP: center of pressure; FL: left front leg; FR: right front leg; HL: left hind leg, HR: right hind leg; MLD: mediolateral displacement of the COP; SS: Support surface; blue thin line: statokinesiogram, path of the COP; graph adapted from^21^.

**Fig. 3:**
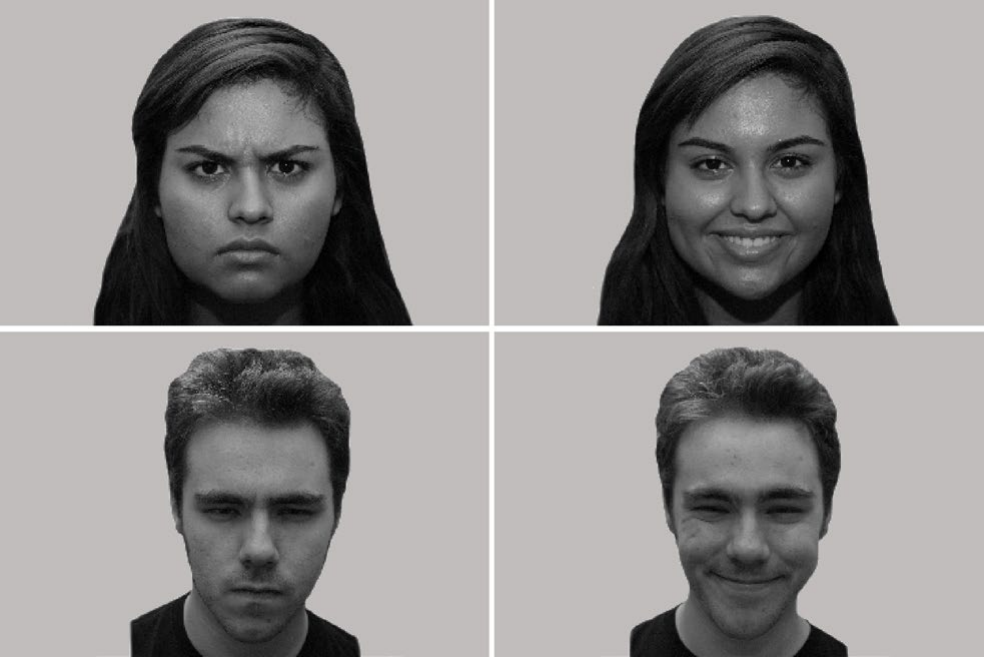
Boxplots depicting the mean, median, interquartile range, and minimum and maximum values for all 5 evaluated ΔCOP_% parameters based on the cluster analysis expressed in percent difference to the no-picture condition. ΔMLD_%: mediolateral displacement; ΔCCD_%: craniocaudal displacement; ΔL_%: length of the COP; ΔAS: average speed of the COP; ΔSS_%: support surface; Δ: individual dog reaction expressed as percent difference when compared to the no-picture condition; blue boxplots: data of all dogs classed to cluster number 1; orange boxplots: data of all dogs classed into cluster 2; angry: viewing angry human pictures; happy: viewing happy human pictures; outliers are indicated by a circle (1 outlier- ΔSS_% dog 2, angry picture 186.4%- not displayed to ensure graphical comparability with the x-axis set at a maximum upper limit of 130%).

All dogs were positioned facing the screen with their front feet approx. 0.75–1.00 m away^7,54^ by using positive reinforcement methods. The height of the screen was individually adjusted using interchangeable opaque boxes, positioning each dog’s eyes approximately at the center of the screen. Then the dog handler asked the dog to wait, walked behind the screen, turned around and kneeled, while keeping invisible and silent throughout the trial (for a schematic set up see Fig. 2). For a valid trial the dogs had to remain standing still on the pressure platform with all limbs perpendicular to the platform when looking at the three different visual conditions. Data were discarded by the observer if any body, head (including turning and tilting the head), tail, limb, or paw movements were observed on the video recording (Fig. 3).

### Data analysis

The fractional lifespan (FLS)^55^ was calculated using the following formula adjusted from imperial to metric units:

$$\:\mathrm{F}\mathrm{L}\mathrm{S}=\:13.620\:+\:\left(0.0276\:\mathrm{b}\mathrm{o}\mathrm{d}\mathrm{y}\:\times\:\:\mathrm{h}\mathrm{e}\mathrm{i}\mathrm{g}\mathrm{h}\mathrm{t}\:\mathrm{i}\mathrm{n}\:\mathrm{c}\mathrm{m}\right)-\:\left(0.1186\:\times\:\:\mathrm{b}\mathrm{o}\mathrm{d}\mathrm{y}\:\mathrm{m}\mathrm{a}\mathrm{s}\mathrm{s}\:\mathrm{i}\mathrm{n}\:\mathrm{k}\mathrm{g}\right)$$

All data were analyzed with the custom-made software Pressure Analyzer (Michael Schwanda, version 4.8.7.0), and exported to Excel (Microsoft, Excel, 2016) for further analysis.

The following parameters were used for the evaluation of the inclusion criteria:

- The mean speed (m/s) and acceleration (m/s²) were calculated for the left forelimb.
- Symmetry index (SI) expressed as a percentage (SI%), was calculated for both parameters (PFz and IFz) according to the following modified equation^53^:

$$\:\mathrm{S}\mathrm{I}\mathrm{X}\mathrm{F}\mathrm{z}\:\left(\mathrm{\%}\right)=\mathrm{a}\mathrm{b}\mathrm{s}\:\left(\right[\:\mathrm{X}\mathrm{F}\mathrm{z}\mathrm{L}\mathrm{L}\mathrm{x}\:\--\:\mathrm{X}\mathrm{F}\mathrm{z}\mathrm{R}\mathrm{L}\mathrm{x}\:]\:/[\mathrm{X}\mathrm{F}\mathrm{z}\mathrm{L}\mathrm{L}\mathrm{x}\:+\:\mathrm{X}\mathrm{F}\mathrm{z}\mathrm{R}\mathrm{L}\mathrm{x}\left]\right)\:\mathrm{x}\:100\:$$

where XFz is the mean value of PFz or IFz of valid steps, LLx is the left front or hindlimb, and RLx is the right front or hind; perfect symmetry between the right and left front or hindlimbs was assigned a value of 0%.

For posturographic analysis, all data were low-pass filtered using a fourth-order Butterworth Filter with a cutoff frequency of 10 Hz^56^. The following parameters were analyzed:

- Base of support (BOS): area enclosed by the coordinates of the center of the paws in square centimeters (cm^2^).
- Base of support length (BOS L): distance between the center of the front and hindlimbs in centimeters (cm).
- Base of support width (BOS W): distance between the center of the left and right limbs in centimeters (cm).
- Craniocaudal displacement: deviation on the craniocaudal axis in millimeters (mm). It was normalized to the BOS L and expressed as a percentage (CCD_%).
- Mediolateral displacement: deviation on the lateral axis millimeters (mm). It was normalized to the BOS W and expressed as a percentage (MLD_%).
- Statokinesiogram length: the length of the line that joins the points of the COP trajectory in meters (m). It was normalized to the BOS and expressed as a percentage (L_%).
- Support surface or statokinesiogram: The area determined by an ellipse that contains 90% of the points of the COP trajectory in mm^2^. It was normalized to the BOS and expressed as a percentage (SS_%).
- Mean speed (AS) (mm/s) of COP sway.

Based on a validity study of static posturography measurements in dogs, the mean COP values from two valid trials of 5 s for each visual condition were calculated and used for further analysis for each dog^57^.

For statistical analysis IBM SPSS version 29 (IBM, Chicago, USA) was used. Descriptive statistics were calculated for all COP data. Data was analyzed for normality assumption using the Shapiro-Wilk test. To determine significant differences between visual condition (no-picture, happy, angry), a multivariate ANOVA test was carried out, including a Bonferroni post hoc test, employing a statistical significance level set at *p* < 0.05.

Based on multiple canine behaviour studies it is known that individual dogs can react differently to social and non-social stimuli including behavioural responses such as freeze, flight and fidget^25–27^. To assess these different behavioural strategies to the presented visual stimuli, individual reactions were calculated for each dog and all COP parameters and expressed as percent increase or decrease where the COP values of the no-picture condition served as baseline. Individual percentage reactions were calculated for both visual conditions (angry and happy) separately.

Individual percentage COP reactions were expressed as ΔMLD_%, ΔCCD_%, ΔL_%, ΔAS, ΔSS_% using the following formula exemplarily shown for ΔMLD_%:

$$\:\varDelta\:MLD\:\%=\frac{MLD\_\%\:\left(human\:picture\:condition\right)-MLD\_\%\:\left(no\:picture\:condition\right)}{MLD\_\%\:\left(no\:picture\:condition\right)}x\:100$$

In addition, all caretakers were asked to fill in a PANAS questionnaire for their dogs. This questionnaire is specifically developed and validated to assesses emotional states by quantifying positive and negative affective traits, serving as indicators of each dog’s temperament^24^.

To classify individual dog reactions cluster analyses were conducted, grouping all COP data based on their relative similarity. A two-stage approach was conducted. The initial step involved a Hierarchical Cluster analysis using Ward’s method and squared Euclidian distances. Based on the visual inspection of the resulting dendrogram, a K-means Cluster analysis was subsequently performed to partition the data into distinct clusters. An ANOVA was then performed to evaluate statistically significant differences in all COP parameters both within and between the identified clusters.

## Supplementary Information

Below is the link to the electronic supplementary material.

Supplementary Material 1

## Data Availability

The raw data supporting the findings of this article will be made available by the authors, on reasonable request by contacting the corresponding author (nadja.affenzeller@vetmeduni.ac.at).
