# Supplementary material for: Happy and angry human pictures differentially affect dogs’ postural stability
Source: Sci Rep. 2026 Feb 3;16:7103. doi: 10.1038/s41598-026-37571-2 (PMC12921248; doi:10.1038/s41598-026-37571-2)
Supplement: Supplementary file 1 — Supplementary Material 1 [file 41598_2026_37571_MOESM1_ESM.docx]

**Table S1 Individual dog data for PANAS score, trial number and human picture per analysed condition for data evaluation in each valid trial.**

| Dog | PANAS (+) | PANAS (-) | Condition | trial number | picture |
| --- | --- | --- | --- | --- | --- |
| 1 | 0.80 | 0.64 | happy 1 | 1 | female |
|  |  |  | happy 2 | 2 | female |
|  |  |  | angry 1 | 1 | male |
|  |  |  | angry 2 | 1 | female |
| 2 | 0.66 | 0.49 | happy 1 | 2 | male |
|  |  |  | happy 2 | 3 | female |
|  |  |  | angry 1 | 1 | female |
|  |  |  | angry 2 | 3 | female |
| 3 | 0.66 | 0.53 | happy 1 | 1 | female |
|  |  |  | happy 2 | 2 | female |
|  |  |  | angry 1 | 1 | male |
|  |  |  | angry 2 | 1 | female |
| 4 | 0.78 | 0.27 | happy 1 | 1 | male |
|  |  |  | happy 2 | 3 | female |
|  |  |  | angry 1 | 1 | male |
|  |  |  | angry 2 | 1 | female |
| 5 | 0.76 | 0.24 | happy 1 | 1 | female |
|  |  |  | happy 2 | 1 | male |
|  |  |  | angry 1 | 1 | male |
|  |  |  | angry 2 | 2 | female |
| 6 | 0.69 | 0.35 | happy 1 | 1 | male |
|  |  |  | happy 2 | 2 | female |
|  |  |  | angry 1 | 1 | female |
|  |  |  | angry 2 | 2 | female |
| 7 | 0.72 | 0.24 | happy 1 | 1 | female |
|  |  |  | happy 2 | 1 | male |
|  |  |  | angry 1 | 1 | female |
|  |  |  | angry 2 | 2 | male |
| 8 | 0.80 | 0.36 | happy 1 | 1 | female |
|  |  |  | happy 2 | 3 | female |
|  |  |  | angry 1 | 3 | male |
|  |  |  | angry 2 | 2 | male |
| 9 | 0.76 | 0.25 | happy 1 | 1 | male |
|  |  |  | happy 2 | 2 | male |
|  |  |  | angry 1 | 1 | female |
|  |  |  | angry 2 | 2 | female |
| 10 | 0.86 | 0.38 | happy 1 | 1 | male |
|  |  |  | happy 2 | 3 | male |
|  |  |  | angry 1 | 1 | female |
|  |  |  | angry 2 | 3 | male |
| 11 | 0.72 | 0.62 | happy 1 | 1 | male |
|  |  |  | happy 2 | 2 | male |
|  |  |  | angry 1 | 1 | male |
|  |  |  | angry 2 | 2 | male |
| 12 | 0.76 | 0.76 | happy 1 | 1 | female |
|  |  |  | happy 2 | 2 | female |
|  |  |  | angry 1 | 1 | female |
|  |  |  | angry 2 | 2 | female |
| 13 | 0.68 | 0.35 | happy 1 | 1 | female |
|  |  |  | happy 2 | 1 | male |
|  |  |  | angry 1 | 1 | male |
|  |  |  | angry 2 | 1 | female |
| 14 | 0.74 | 0.49 | happy 1 | 1 | female |
|  |  |  | happy 2 | 1 | male |
|  |  |  | angry 1 | 1 | male |
|  |  |  | angry 2 | 1 | female |
| 15 | 0.84 | 0.56 | happy 1 | 1 | male |
|  |  |  | happy 2 | 3 | female |
|  |  |  | angry 1 | 1 | male |
|  |  |  | angry 2 | 1 | female |
| 16 | 0.74 | 0.25 | happy 1 | 2 | female |
|  |  |  | happy 2 | 3 | male |
|  |  |  | angry 1 | 1 | female |
|  |  |  | angry 2 | 2 | male |
| 17 | 0.67 | 0.42 | happy 1 | 1 | female |
|  |  |  | happy 2 | 1 | male |
|  |  |  | angry 1 | 1 | male |
|  |  |  | angry 2 | 1 | female |

PANAS (+): positive activation score of each individual dog; PANAS (-): negative activation score of each individual dog; happy 1,2: happy human picture presentation, all further calculations were based on data mean of both happy human picture presentation; angry: angry human picture presentation, all further calculations were based on data mean of both angry human picture presentation; trial number 1-3: trial number from which data were taken and further analysed; picture: indicating the human picture gender (female, male) from which data were further analysed; n.d. not determined, owner lost for follow up

**Table S2: Descriptive statistics of all non-normalized COP parameters (before normalization based on base of support data) in different visual conditions**

| Condition | MLD | CCD | L | AS | SS |
| --- | --- | --- | --- | --- | --- |
| No-pic | 2.46 ± 0.72 | 6.35 ± 1.72 | 0.09 ± 0.03 | 20.43 ± 6.83 | 9.12 ± 4.28 |
| Happy | 2.62 ± 1.00 | 5.81 ± 1.40 | 0.09 ± 0.03 | 18.56 ± 4.09 | 8.66 ± 4.42 |
| Angry | 2.90 ± 1.26 | 6.18 ± 1.97 | 0.09 ± 0.03 | 17.94 ± 4.40 | 10.24 ± 5.53 |

MLD: mediolateral displacement in mm; CCD: craniocaudal displacement in mm; L: length of the COP in m; AS: average speed of the COP in mm/s; SS: support surface in mm^2^; ±: standard deviation; no-pic: no picture presentation; happy: happy human picture presentation; angry: angry human picture presentation

**Table S3**: **Descriptive statistics of all COP parameters and cluster number in different visual conditions per individual dog.**

| Dog | Condition | MLD_% | CCD_% | L_% | AS | SS_% | ΔMLD_% | ΔCCD_% | ΔL_% | ΔAS | ΔSS_% | Cluster |
| --- | --- | --- | --- | --- | --- | --- | --- | --- | --- | --- | --- | --- |
| 1 | No-pic | 1.22 | 1.34 | 0.10 | 16.34 | 0.08 |  |  |  |  |  |  |
|  | Happy | 1.06 | 1.24 | 0.09 | 17.45 | 0.07 | -13.4 | -7.3 | -7.2 | 6.8 | -12.5 | 1 |
|  | Angry | 2.28 | 1.54 | 0.11 | 18.42 | 0.07 | 86.9 | 14.6 | 6.3 | 12.7 | -12.5 | 2 |
| 2 | No-pic | 0.81 | 0.92 | 0.08 | 19.05 | 0.05 |  |  |  |  |  |  |
|  | Happy | 0.85 | 0.93 | 0.08 | 14.43 | 0.05 | 4.4 | 0.2 | -9.7 | -24.2 | 0.5 | 1 |
|  | Angry | 1.11 | 1.63 | 0.08 | 15.46 | 0.13 | 36.3 | 76.1 | -1.4 | -18.8 | 186.4 | 2 |
| 3 | No-pic | 0.73 | 0.86 | 0.07 | 17.51 | 0.04 |  |  |  |  |  |  |
|  | Happy | 1.26 | 1.04 | 0.07 | 17.90 | 0.10 | 72.3 | 21.3 | -5.7 | 2.2 | 150.0 | 2 |
|  | Angry | 1.44 | 1.06 | 0.08 | 22.88 | 0.10 | 97.1 | 23.1 | 14.0 | 30.7 | 150.0 | 2 |
| 4 | No-pic | 1.40 | 1.26 | 0.11 | 32.62 | 0.05 |  |  |  |  |  |  |
|  | Happy | 1.09 | 0.94 | 0.08 | 23.78 | 0.06 | -21.8 | -25.6 | -26.6 | -27.1 | 22.7 | 1 |
|  | Angry | 0.89 | 1.74 | 0.07 | 24.01 | 0.06 | -36.3 | 38.3 | -31.8 | -26.4 | 17.8 | 1 |
| 5 | No-pic | 1.13 | 1.03 | 0.09 | 17.15 | 0.10 |  |  |  |  |  |  |
|  | Happy | 1.15 | 0.99 | 0.10 | 18.88 | 0.11 | 1.6 | -3.7 | 12.8 | 10.1 | 14.9 | 1 |
|  | Angry | 1.32 | 1.13 | 0.09 | 16.86 | 0.10 | 16.9 | 10.0 | -3.5 | -1.7 | 4.5 | 2 |
| 6 | No-pic | 1.00 | 0.67 | 0.07 | 11.55 | 0.05 |  |  |  |  |  |  |
|  | Happy | 2.23 | 0.79 | 0.08 | 14.18 | 0.08 | 122.8 | 17.7 | 11.2 | 22.8 | 54.0 | 2 |
|  | Angry | 2.15 | 0.85 | 0.05 | 10.70 | 0.07 | 115.2 | 26.4 | -22.2 | -7.3 | 48.6 | 2 |
| 7 | No-pic | 1.08 | 1.02 | 0.07 | 14.45 | 0.07 |  |  |  |  |  |  |
|  | Happy | 0.94 | 1.04 | 0.09 | 22.95 | 0.06 | -13.3 | 2.0 | 25.5 | 58.8 | -12.6 | 1 |
|  | Angry | 1.53 | 1.42 | 0.07 | 17.27 | 0.12 | 41.7 | 39.3 | -1.5 | 19.5 | 67.1 | 2 |
| 8 | No-pic | 1.29 | 1.28 | 0.10 | 16.56 | 0.09 |  |  |  |  |  |  |
|  | Happy | 1.38 | 1.15 | 0.07 | 13.37 | 0.11 | 7.2 | -10.5 | -26.8 | -19.3 | 21.2 | 1 |
|  | Angry | 0.99 | 0.72 | 0.06 | 12.88 | 0.04 | -23.2 | -44.1 | -37.7 | -22.2 | -56.4 | 1 |
| 9 | No-pic | 1.27 | 1.35 | 0.06 | 15.70 | 0.10 |  |  |  |  |  |  |
|  | Happy | 0.86 | 0.83 | 0.05 | 15.86 | 0.04 | -31.9 | -38.8 | -11.5 | 1.1 | -56.1 | 1 |
|  | Angry | 0.64 | 0.71 | 0.05 | 13.48 | 0.04 | -49.5 | -47.1 | -17.8 | -14.1 | -64.7 | 1 |
| 10 | No-pic | 1.82 | 1.52 | 0.11 | 19.58 | 0.18 |  |  |  |  |  |  |
|  | Happy | 1.36 | 1.21 | 0.10 | 23.18 | 0.11 | -25.2 | -20.4 | -8.9 | 18.4 | -39.2 | 1 |
|  | Angry | 1.30 | 1.11 | 0.09 | 20.96 | 0.10 | -28.5 | -26.8 | -18.0 | 7.1 | -47.2 | 1 |
| 11 | No-pic | 1.98 | 1.19 | 0.16 | 33.59 | 0.12 |  |  |  |  |  |  |
|  | Happy | 0.91 | 0.81 | 0.07 | 12.77 | 0.04 | -53.8 | -32.1 | -57.0 | -62.0 | -67.7 | 1 |
|  | Angry | 0.82 | 0.76 | 0.07 | 12.09 | 0.04 | -58.7 | -36.0 | -56.4 | -64.0 | -65.0 | 1 |
| 12 | No-pic | 1.26 | 1.37 | 0.15 | 31.47 | 0.10 |  |  |  |  |  |  |
|  | Happy | 1.30 | 0.84 | 0.08 | 20.87 | 0.07 | 3.3 | -38.4 | -49.8 | -33.7 | -27.3 | 1 |
|  | Angry | 1.59 | 1.14 | 0.08 | 21.50 | 0.11 | 25.9 | -16.5 | -46.1 | -31.7 | 12.7 | 1 |
| 13 | No-pic | 1.54 | 1.67 | 0.08 | 16.15 | 0.09 |  |  |  |  |  |  |
|  | Happy | 1.76 | 1.40 | 0.08 | 16.62 | 0.15 | 14.0 | -16.1 | 5.7 | 2.9 | 70.7 | 2 |
|  | Angry | 1.43 | 1.92 | 0.08 | 17.28 | 0.17 | -7.4 | 14.7 | 4.2 | 7.0 | 91.0 | 2 |
| 14 | No-pic | 1.04 | 1.22 | 0.07 | 13.87 | 0.07 |  |  |  |  |  |  |
|  | Happy | 1.20 | 1.51 | 0.11 | 16.78 | 0.11 | 15.6 | 23.7 | 64.1 | 21.0 | 63.6 | 2 |
|  | Angry | 1.41 | 1.24 | 0.09 | 14.06 | 0.09 | 35.4 | 1.3 | 35.2 | 1.3 | 34.2 | 2 |
| 15 | No-pic | 1.40 | 1.03 | 0.07 | 21.01 | 0.08 |  |  |  |  |  |  |
|  | Happy | 1.54 | 1.32 | 0.06 | 17.62 | 0.12 | 10.3 | 28.2 | -7.6 | -16.2 | 48.1 | 2 |
|  | Angry | 1.56 | 1.02 | 0.07 | 19.79 | 0.10 | 11.7 | -1.0 | 4.5 | -5.8 | 23.1 | 1 |
| 16 | No-pic | 0.90 | 0.91 | 0.09 | 24.52 | 0.06 |  |  |  |  |  |  |
|  | Happy | 1.57 | 1.26 | 0.09 | 21.83 | 0.08 | 74.9 | 38.9 | 4.4 | -11.0 | 34.6 | 2 |
|  | Angry | 1.04 | 1.02 | 0.09 | 22.67 | 0.06 | 15.4 | 11.9 | 5.2 | -7.5 | 3.0 | 1 |
| 17 | No-pic | 1.38 | 1.03 | 0.13 | 26.19 | 0.08 |  |  |  |  |  |  |
|  | Happy | 1.05 | 1.06 | 0.10 | 26.98 | 0.06 | -24.2 | 2.9 | -21.8 | 3.0 | -27.6 | 1 |
|  | Angry | 1.23 | 1.06 | 0.10 | 24.72 | 0.08 | -10.6 | 3.1 | -23.5 | -5.6 | -4.1 | 1 |
| Minimum |  |  |  |  |  |  | -58.7 | -47.1 | -57.0 | -64.0 | -67.7 |  |
| Maximum |  |  |  |  |  |  | 122.8 | 76.1 | 64.1 | 58.8 | 186.4 |  |
| %_difference range |  |  |  |  |  |  | 181.5 | 123.1 | 121.2 | 122.8 | 254.1 |  |

MLD_%: mediolateral displacement; CCD %: craniocaudal displacement; L_%: length of the COP; AS: average speed of the COP; SS_%: support surface; Δ: individual dog reaction expressed as percent difference when compared to the no_picture condition for every COP parameter; Cluster: cluster number of each dog based on cluster analysis; Happy: viewing happy human pictures; Angry: viewing angry human pictures; no_pic: viewing no picture; Minimum %: minimum Δ difference; Maximum %: maximal Δ difference; Δ_range: difference between maximum and minimum Δ differences
